# Long noncoding RNA as an emerging regulator of endoderm differentiation: progress and perspectives

**DOI:** 10.1186/s13619-025-00230-4

**Published:** 2025-03-26

**Authors:** Jie Yang, Donghui Zhang, Wei Jiang

**Affiliations:** 1 State Key Laboratory of Biocatalysis and Enzyme Engineering, School of Life Sciences, Hubei University, Wuhan, 430062 China; 2 Department of Biological Repositories, Frontier Science Center for Immunology and Metabolism, Medical Research Institute, Zhongnan Hospital of Wuhan University, Wuhan University, Wuhan, 430071 China; 3 Hubei Provincial Key Laboratory of Developmentally Originated Disease, Wuhan, 430071 China

**Keywords:** LncRNA, Endoderm differentiation, Pluripotent stem cell, Proximal lncRNA, Desert lncRNA

## Abstract

Accumulated studies have demonstrated that long noncoding RNAs (lncRNAs) play crucial regulatory roles in diverse biological processes, such as embryonic development and cell differentiation. Comprehensive transcriptome analysis identifies extensive lncRNAs, gradually elucidating their functions across various contexts. Recent studies have highlighted the essential role of lncRNAs in definitive endoderm differentiation, underscoring their importance in early development. In this review, we have analyzed the features of overlapping, proximal, and desert lncRNAs, classified by genomic location, in pluripotent stem cells (PSCs) and the differentiation derivatives. Furthermore, we focus on the endoderm lineage and review the latest advancements in lncRNA identification and their distinct regulatory mechanisms. By consolidating current knowledge, we aim to provide a clearer perspective on how lncRNAs contribute to endoderm differentiation in different manners.

## Background

Definitive endoderm arises from the epiblast originated from the inner cell mass (ICM), and further differentiates to form the organs and tissues of the digestive and respiratory systems. Aberrant development of the endoderm can lead to severe developmental diseases such as congenital pulmonary hypoplasia, congenital biliary atresia, and pancreatic and hepatic dysplasia. In vitro endoderm differentiation from embryonic stem cells (ESCs) or induced pluripotent stem cells (iPSCs) offers a powerful approach to exploring the regulatory mechanisms governing definitive endoderm development and its derivatives. Multifaceted regulatory processes, including key signaling pathways, metabolic and epigenetic regulation orchestrate the development of definitive endoderm (Fang and Li 2022; Yang and Jiang 2020). As omics studies advance, increasing evidence highlights the significant roles of noncoding RNAs in regulating biological processes. Besides microRNAs (Chen and Kim 2024), lncRNAs stand out due to their diverse regulatory modes and functions.

LncRNAs are RNA molecules exceeding 200 nucleotides in length and lacking evident protein-coding potential. LncRNA can play a crucial role in X-chromosome inactivation (Marahrens et al. 1998; Penny et al. 1996), and actively participate in cell differentiation and development (Fatica and Bozzoni 2014) as well as cancers (Schmitt and Chang 2016). A genome-wide CRISPR interference (CRISPRi) screening targeting 16,401 lncRNA loci across seven diverse cell lines identified that 449 lncRNAs are essential for robust cellular growth; perturbations of these lncRNAs disrupt transcription networks in a cell-type-specific manner (Liu et al. 2017). In human ESCs and differentiated endoderm and mesoderm cells, approximately 12,611 lncRNA transcripts are expressed in at least one of the three cell types while 4,158 are differentially expressed between human ESCs and the derivatives (Haswell et al. 2021).

With the advancement of sequencing technology and the growing scientists on lncRNA studies, it has become evident that lncRNA transcripts are expressed in diverse contexts and play intricate roles through various regulatory mechanisms. To gain deeper insights into their functions and regulatory models, it is essential to systematically characterize their features and roles in specific cellular processes. In this review, we focus on endoderm differentiation, summarizing the characteristics and functions of lncRNAs based on their classification. Drawing from previous studies, we refine their classification into three categories: overlapped, proximal, and desert lncRNAs, according to their genomic distance from neighboring protein-coding genes (PCGs). We then characterize these subgroups in terms of protein-coding potential, expression levels, subcellular localization, and expression specificity. Furthermore, we explore the roles and regulatory mechanisms of different lncRNA subgroups in endoderm differentiation, highlighting their functional diversity. Finally, we discuss the challenges associated with studying lncRNA functions and mechanisms and the future research directions. This review provides a comprehensive perspective on the classification and functional roles of lncRNAs in endoderm differentiation, offering valuable insights into their regulatory complexity and biological significance.

## Characteristics and classification of lncRNAs

The recently updated human GENCODE v46 annotation encompasses 63,086 genes and 254,070 transcripts, including 19,258 lncRNA genes and 20,065 protein-coding genes (PCGs) (Frankish et al. 2023). The substantial number of lncRNAs underscores their essential role in human development, prompting increased research into their functions across various biological processes. Due to their vast quantity and intricate regulatory models, lncRNAs are categorized mainly based on genomic locations. In the human GENCODE v7 catalog, lncRNAs are classified into “genic” and “intergenic” categories based on whether they intersect with protein-coding loci. Genic lncRNAs are further divided into intronic and exonic types, with these subcategories further subdivided according to their transcription orientation. Intergenic lncRNAs are classified into three groups based on their transcription orientation: the same strand, convergent, and divergent (Derrien et al. 2012). Similarly, another group differentiates lncRNA as genic (within 5 kb of a PCG) and intergenic, with genic lncRNAs further subdivided into divergent, convergent, antisense inside, antisense outside, sense downstream, and sense upstream categories. Divergent and antisense-inside lncRNAs represent the two largest genic lncRNA biotypes in human and mouse genomes. Antisense lncRNAs are more likely to be co-expressed with nearby genes than others (Luo et al. 2016). In addition, lncRNA can also be classified in other ways, such as lncRNA function, lncRNA length, lncRNA biogenesis pathways, lncRNA subcellular localization, and so on (Jarroux et al. 2017; Kopp and Mendell 2018). While these classifications help in understanding the detailed characteristics of lncRNAs, they also complicate the study of their functions and mechanisms. Additionally, the features of different lncRNA groups are not systematically characterized.

**Fig. 1:**
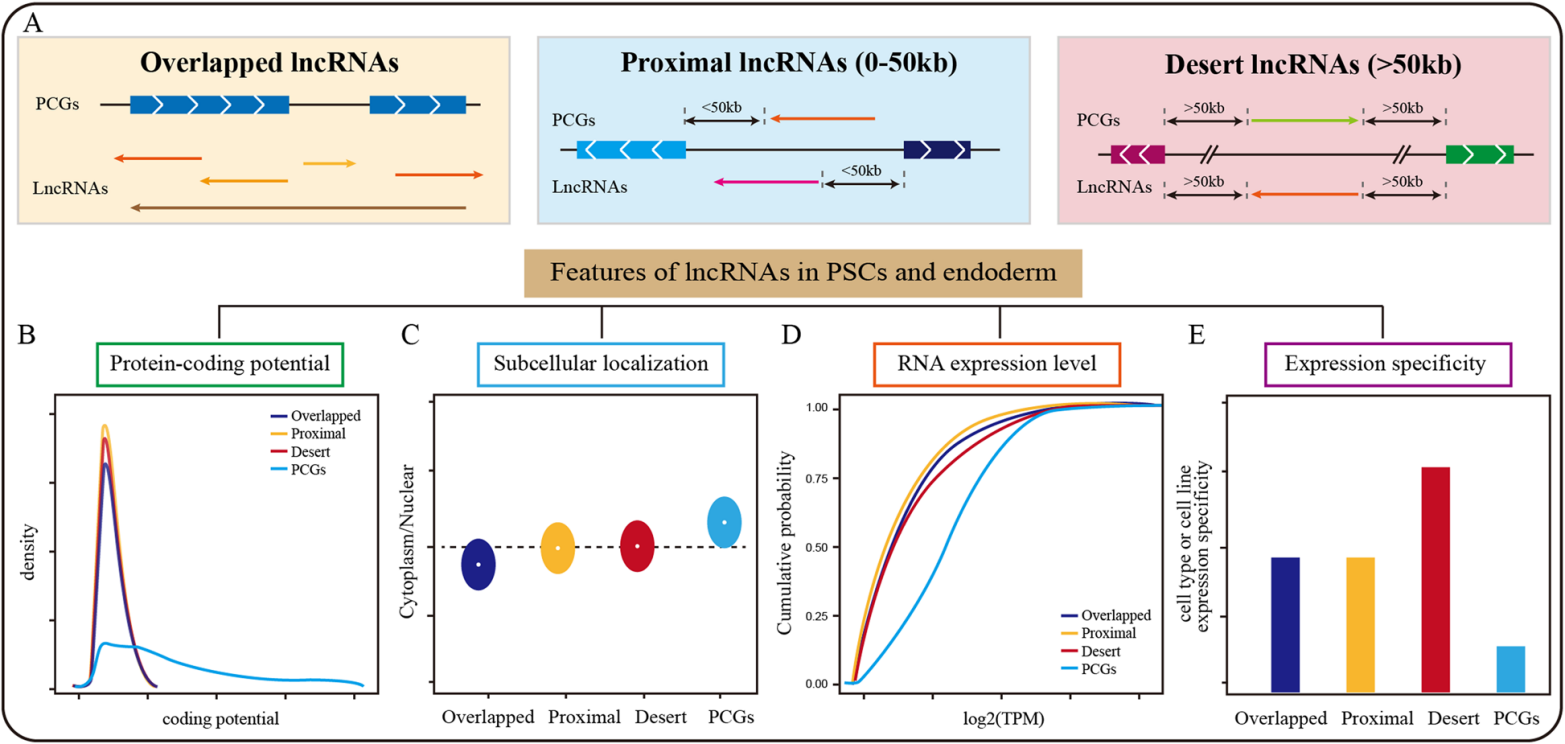
The classification and features of lncRNAs in PSCs and endoderm cells. **A**. Based on their genomic locations, lncRNAs can be classified into overlapping lncRNAs, proximal lncRNAs, and desert lncRNAs. **B**-**E**. The characteristics of different lncRNA subtypes in PSCs and endoderm cells are summarized primarily from reference (Lu et al. 2023). Compared to PCGs, the three types of lncRNAs generally exhibit lower coding potential (**B**) and expression levels (**C**), yet they show a stronger tendency for nuclear localization (**D**). To quantify the stage-specificity of genes and lncRNAs, we apply a stage-specificity score (score = meanA-(meanOther + 2 × sdOther)) (Jiang et al. 2015) and observe that lncRNAs, particularly desert lncRNAs, exhibit stronger cell-type-specific expression compared to PCGs (**E**)

Recently, we proposed a simplified classification by integrating lncRNA location and cis-/trans-regulatory models, dividing lncRNAs into three categories (Lu et al. 2023): overlapped lncRNAs (sharing at least one nucleotide with PCGs), proximal lncRNAs (within 50 kb of PCGs but non-overlapping), and desert lncRNAs (more than 50 kb away from the nearest PCGs) (Fig. 1A). This classification provides a streamlined framework to study lncRNAs by integrating their genomic location and regulatory modes, making it easier for new comers to identify their targets and select appropriate methods for investigating lncRNA mechanisms. Notably, overlapped lncRNAs constitute over half of the total. Analysis in PSCs and endoderm cells reveals that lncRNA generally exhibits lower protein-coding potential and a stronger inclination toward nuclear localization, particularly among overlapped lncRNAs (Lu et al. 2023) (Fig. 1B, C). This low coding potential indicates that most lncRNAs likely perform biological functions primarily through their transcripts, with only a few able to encode small peptides (Choi et al. 2019). The strong nuclear localization tendency of lncRNAs may be partly due to their degradation by the nuclear surveillance machinery (Schlackow et al. 2017; Tuck and Tollervey 2013). On the other hand, nuclear-retained stable lncRNAs play key roles in regulating chromatin state, transcription and nuclear organization (Statello et al. 2021; Yin et al. 2020). Compared to protein-coding genes, lncRNAs typically have lower expression levels; however, desert lncRNAs are apparently higher than the other two sub-groups (Fig. 1D). All lncRNA types exhibit a more excellent cell type and lineage-specific expression pattern than protein-coding genes (Haswell et al. 2021; Lu et al. 2023) (Fig. 1E). Consistently, the comprehensive analysis of lncRNAs in multiple human organs/tissues and brain regions reveals that lncRNAs are generally lower expressed and more tissue-specific than PCGs (Cabili et al. 2011; Derrien et al. 2012). Taken together, the lncRNA’s classification and characteristic analysis support their critical regulatory roles in development and transcription, necessitating a nuanced understanding of their diverse categories and expression profiles.

## Regulatory role of lncRNAs in human endoderm differentiation

### Overlapped lncRNAs regulate endoderm differentiation: action *in cis* versus *trans*

**Fig. 2:**
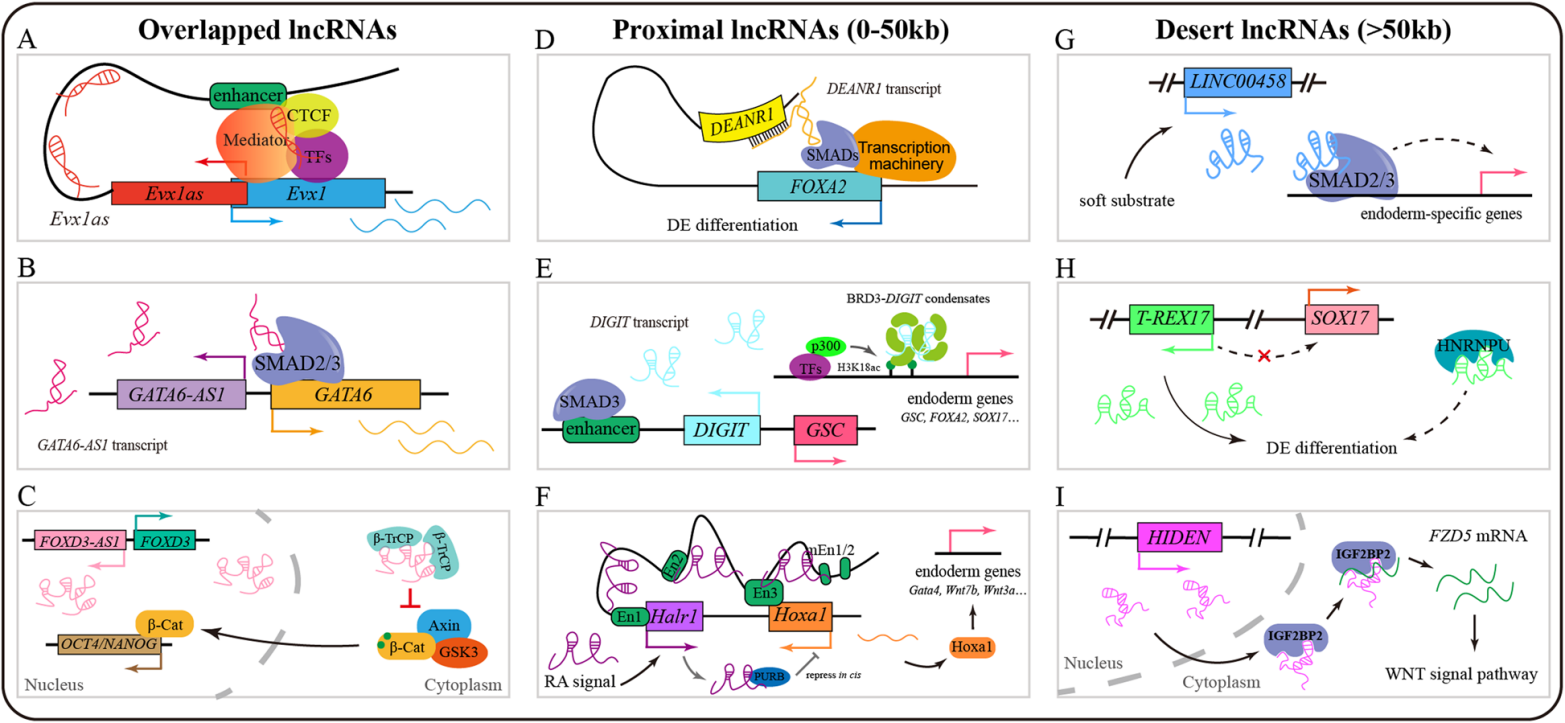
The functions and mechanisms of lncRNAs in endoderm differentiation. **A**. The overlapped lncRNAs: *Evxas1* is transcribed and binds locally to chromatin, facilitating the recruitment of the transcriptional coactivator Mediator, CTCF, and transcription factors (TFs), and thus establishing an open chromatin configuration. The local chromatin changes enhance the interaction between the *Evx1* promoter and its enhancer, activating *Evx1* transcription and orchestrating the regulation of endoderm differentiation. **B**. *GATA6-AS1* interacts with SMAD2/3 and facilitates their recruitment to the *GATA6* promoter, activating *GATA6* transcription and promoting endoderm differentiation. **C**. *FOXD3-AS1* functions in the cytoplasm by interacting with β-TrCP, thereby blocking its interaction with Axin/GSK, which phosphorylates β-catenin and leads to β-catenin degradation. As a result, unphosphorylated β-catenin translocates into the nucleus, regulating the expression of essential pluripotency genes such as *OCT4* and *NANOG*, but with no effect on the expression of proximal gene *FOXD3*. **D**. The proximal lncRNAs: *DEANR1* interacts with SMAD2/3 and facilitates their recruitment to the *FOXA2* promoter, potentially through chromatin looping. In conjunction with other transcriptional machinery components, it promotes *FOXA2* transcription in endoderm differentiation. **E**. SMAD3 binds to the proximal enhancer during endoderm differentiation, activating *DIGIT* transcription. *DIGIT* interacts with BRD3, promoting the formation of phase-separated BRD3 condensates. Simultaneously, *DIGIT* recruits BRD3- *DIGIT* condensates to H3K18ac regions, inducing the expression of crucial endoderm genes. **F**. *Halr1* is induced by RA signaling and functions as a scaffold, facilitating the interaction between *Hoxa1* chromatin and enhancer En3, thereby maintaining the overall chromatin structure of the *Hoxa* cluster. Additionally, *Halr1* suppresses *Hoxa1* expression as a brake by blocking its chromatin from interacting with enhancers En1, En2, and mini-enhancers. On the other hand, Halr1 can interact with PURB to repress Hoxa1 expression *in cis*. **G**. The desert lncRNAs: *LINC00458* is upregulated upon soft substrate and interacts with SMAD2/3, facilitating endoderm gene expression by unclear mechanism. **H**. The lncRNA *T-REX17* is activated by SOX17 and interacts with HNRNPU. However, its regulatory mechanism in endoderm differentiation remains unknown. **I**. The desert lncRNA *HIDEN* interacts with IGF2BP1 to regulate the stability of *FZD5* mRNA, thereby activating the WNT signaling pathway, which plays a critical role in endoderm differentiation.

In ESCs and derived endoderm cells, a significant proportion of lncRNAs are transcribed divergently from the promoters of active PCGs. Notable examples include lncRNA *Evx1as* and lncRNA *GATA6-AS1* (Luo et al. 2016; Sigova et al. 2013). The divergent lncRNA *Evx1as* overlaps with the neighboring coding gene *Evx1*, a homeodomain transcription factor that promotes mesoderm differentiation (Kalisz et al. 2012). Knockdown of *Evx1as* diminishes the activation of *Evx1* during the differentiation of mouse ESCs, while *Evx1as*-null mouse ESCs exhibit impaired activation of mesendoderm-related genes. Mechanistically, *Evx1as* transcripts promote chromatin looping by binding to its gene locus, enhancing the interaction between the *Evx1* promoter and a potential enhancer located 3’ downstream of *Evx1as*. Additionally, *Evx1as* RNA binds to its transcription sites and downstream regulatory region on chromatin, interacts with MED1 and MED12, and promotes an active chromatin state. Ultimately, *Evx1as* transcripts may facilitate the binding of the mediator complex, shaping the local chromatin environment to activate *Evx1* and regulate mesendoderm differentiation (Luo et al. 2016) (Fig. 2A).

In the human case, lncRNA locus 5689, also called *GATA6-AS1*, is highly expressed in differentiated endoderm cells (Yang et al. 2020). The expression of *GATA6-AS1* is positively correlated with the neighboring gene *GATA6* during endoderm differentiation. Knockdown of *GATA6-AS1* led to a decreased expression of *GATA6* and thus blocked endoderm differentiation. Meanwhile, transcriptome analysis showed that the deficiency of *GATA6-AS1* caused the downregulation of definitive endoderm signature genes. Further studies indicate that *GATA6-AS1* interacts with TGF-β signal effector SMAD2/3 and promotes SMAD2/3 binds to *GATA6* promoter, modulating human endoderm differentiation (Yang et al. 2020) (Fig. 2B). A subsequent study reports that knockdown of *GATA6-AS1* decreases *GATA6* expression and inhibits mesoderm induction and cardiomyocyte differentiation from human PSCs via regulating the WNT signal pathway (Jha et al. 2020).

During endoderm differentiation from ESCs, a large fraction of lncRNAs arise from divergent transcription of neighboring PCGs and show coordinated expression with PCGs in the nucleus (Sigova et al. 2013). Similarly, in other tissues or cell lines, lncRNAs also exhibit co-expression with nearby PCGs (Cabili et al. 2011; Derrien et al. 2012; Ponjavic et al. 2009), showing the potential of the overlapped lncRNAs regulating the adjacent PCGs *in cis.* However, in some cases, overlapping lncRNAs can also function *in trans*, such as the overlapped lncRNA *FOXD3-AS1*. *FOXD3-AS1* (also named *FAST* in (Guo et al. 2020)) is a positionally conserved lncRNA in human and mouse ESCs. Interestingly, *FOXD3-AS1* shows different subcellular localization and regulation mechanisms between human and mouse ESCs. In human ESCs, cytoplasm-localized *FOXD3-AS1* binds to the WD40 domain of the E3 ubiquitin ligase β-TrCP and blocks its interaction with phosphorylated β-catenin to prevent degradation, leading to activated WNT signaling, an essential pathway for pluripotency maintenance and endoderm differentiation. In contrast, *Foxd3-as1* locates in nuclear and is not required for pluripotency maintenance of mouse ESCs (Guo et al., 2020). Consistently, the genome-wide CRISPR interference screen identified and validated *FOXD3-AS1* as a functional lncRNA essential for pluripotency and differentiation. The knockdown of *FOXD3-AS1* results in losing pluripotency and upregulating several key endoderm factors in human ESCs. Inversely, overexpression of *FOXD3-AS1* represses endoderm factor expression (Haswell et al. 2021) (Fig. 2C).

### Proximal lncRNAs in endoderm differentiation: multilayered transcriptional regulation

Several proximal lncRNAs have also been identified to participate in endoderm differentiation, via different actions. The proximal lncRNA *DEANR1*, also known as *LINC00261*, was reported to regulate human endoderm differentiation in 2015 (Jiang et al. 2015). Located 3' downstream of the endoderm transcription factor *FOXA2*, *DEANR1* expression gradually increases during endoderm differentiation from ESCs. Knockdown of *DEANR1* does not affect the expression of vital pluripotent factors or alter their clonal morphology in human ESCs. However, it significantly decreases endoderm differentiation efficiency and the expression of endoderm marker genes. Notably, *DEANR1* shows a strong correlation with *FOXA2*; its knockdown dramatically reduces *FOXA2* expression, and exogenous *FOXA2* can rescue the differentiation defects caused by *DEANR1* depletion. RNA fluorescent in situ hybridization (FISH) demonstrates that *DEANR1* transcripts are mainly localized to the *FOXA2* gene locus. Further evidence indicates that *DEANR1* interacts with SMAD2/3, recruiting them to the *FOXA2* promoter to regulate endoderm differentiation (Jiang et al. 2015) (Fig. 2D). Beyond its role in endoderm differentiation as a noncoding RNA, another report suggests that *DEANR1* has translation potential and produces microproteins in pancreatic progenitors; however, the functional relevance of these microproteins in endoderm-derived cells remains unclear (Gaertner et al., 2020).

Another proximal lncRNA *DIGIT*, divergently transcribed from *GSC* gene locus, is crucial for both mouse and human endoderm differentiation (Daneshvar et al. 2016). A SMAD2/3-occupied enhancer coordinately regulates *DIGIT* and its neighboring gene *GSC* during endoderm differentiation. Deletion of this enhancer impairs *DIGIT* activation and compromises endoderm differentiation. Depletion of *DIGIT* using short hairpin RNAs (shRNAs), locked nucleic acids (LNAs), or poly(A) termination sequences, demonstrates that the *DIGIT* transcript, rather than transcription activity at the *DIGIT* locus, regulates GSC expression. Interestingly, ectopic expression of *DIGIT* can activate *GSC* expression in *DIGIT*-deficient cells (Daneshvar et al. 2016). In 2020, the same group further elucidated the regulatory mechanism of *DIGIT* in endoderm differentiation, finding that *DIGIT* interacts with the bromodomain and extraterminal domain of BRD3 through interatomic analysis (Daneshvar et al. 2020). BRD3 can form liquid–liquid phase-separated condensates, which are membrane-free biomolecular assemblies that compartmentalize cellular processes. *DIGIT* transcripts promote the formation of these condensates both in vitro and in cells. Similar to the effects of *DIGIT* deficiency, BRD3 depletion also impairs endoderm differentiation. Additionally, BRD3 interacts with acetylated H3K18 (H3K18ac) and co-localizes with genes associated with endoderm differentiation. Consequently, the depletion of *DIGIT* reduces the formation of BRD3 phase-separated condensates, preventing BRD3 from binding to H3K18ac and blocking the activation of endoderm genes (Daneshvar et al. 2020) (Fig. 2E). These results indicate that *DIGIT* can regulate endoderm differentiation both *in cis* and *trans*. This goes beyond the concept that nuclear-localized lncRNAs preferentially regulate nearby PCGs *in cis*.

Additionally, studies have shown that the proximal lncRNA *linc1547* is critical for the maintenance of pluripotency. Knockdown *linc1547* reduces the expression of *Nanog* and *Oct4* (Guttman et al. 2011). Later, Maamar and colleagues revealed the relationship between *linc1547* (referred as *linc-Hoxa1* in their study) and *Hoxa1* through single-molecule RNA imaging and single-cell analysis. They observed a negative correlation between *linc-Hoxa1* and *Hoxa1* expression, which was overridden by retinoic acid (RA) treatment. Interestingly, the knockdown of *linc-Hoxa1* at its transcription site suppresses *Hoxa1* expression, whereas transient expression of *linc-Hoxa1* has no effect on *Hoxa1* levels, suggesting that *linc-Hoxa1* represses *Hoxa1* *in cis*. Further evidence showed that *linc-Hoxa1* exerts this cis-regulatory effect by recruiting the transcriptional cofactor PURB (Maamar et al. 2013) (Fig. 2F).

Interestingly, in 2015, Yin and colleagues identified that *linc1547* (named as *Haunt* in their study) had opposing roles for its RNA transcripts and genomic locus in regulating *Hoxa* genes (Yin et al. 2015). Contrary to earlier findings, they observed that the knockdown of *Haunt* RNA using shRNAs in mouse ESCs does not affect the expression of pluripotency genes or *Hoxa1*. Moreover, depletion of PURB, PURA, or both also has no impact on *Hoxa* expression. However, under RA treatment, reducing *Haunt* RNA levels through RNAi, promoter deletion, or poly(A) insertion increases *Hoxa* expression, revealing a similar negative correlation between *Haunt* RNA and *Hoxa* as before. Using CRISPR/Cas9, the authors created *Haunt* knockout ESCs with genomic deletions ranging from 7.3–58 kb. Interestingly, deletion of large genomic fragments at the *Haunt* locus suppresses *Hoxa* expression, contrasting the effects of *Haunt* RNA knockdown. Further analysis revealed that potential enhancers essential for *Hoxa* activation are located within the *Haunt* genomic locus. These findings suggest that *Haunt* RNA regulates *Hoxa* expression and ESC differentiation by attenuating long-range chromatin interactions between the enhancer within the *Haunt* locus and the *Hoxa* promoter (Yin et al. 2015) (Fig. 2F).

A following report further indicated that *linc1547* (named as *Halr1* in the study) plays a crucial role in enhancer architecture-dependent, multilayered transcriptional regulation at the *Halr1*–*Hoxa1* locus (Su et al. 2021), coordinating RA-induced early lineage differentiation of mouse ESCs. Both *Halr1* and its adjacent gene *Hoxa1* are downstream targets of the RA signaling pathway. Deletion of *Halr1* leads to the overactivation of *Hoxa1* in response to RA signaling, thereby promoting premature endoderm differentiation in ESCs. *Hoxa1* acts as a core transcriptional regulator by directly controlling the expression of a subset of genes involved in endoderm development. Interestingly, a reciprocal regulatory relationship exists between *Hoxa1* and *Halr1*. *Hoxa1* promotes *Halr1* expression in an enhancer-dependent manner, while *Halr1* functions as a scaffold, facilitating the interaction of *Hoxa1* with an enhancer to maintain the chromatin structure of the *HoxA* cluster. Meanwhile, *Halr1* suppresses *Hoxa1* expression by inhibiting chromatin interactions with four distal enhancers, thereby fine-tuning the RA-induced differentiation process (Su et al. 2021) (Fig. 2F).

### Desert lncRNAs interact with regulators to modulate endoderm differentiation

Desert lncRNAs locate in intergenic regions, making their roles in biological processes challenging to predict due to their distance from PCGs. However, recent studies have begun uncovering their regulatory functions in cellular differentiation. Chen and colleagues discovered that a lower substrate stiffness promotes endodermal lineage differentiation. They also observed that several lncRNAs, including *LINC00458*, are upregulated on soft substrates. *LINC00458* is induced within 4 h of exposure to soft substrates and is predominantly localized in the nucleus. The knockdown of *LINC00458* using LNAs and GapmeRs significantly reduces the mRNA and protein levels of the endoderm-specific genes *FOXA2* and *SOX17*, indicating its essential role in the endodermal specification. Further mechanistic studies reveal that *LINC00458* interacts with SMAD2/3 and modulates cell differentiation through a SMAD2/3-dependent pathway (Chen et al. 2020) (Fig. 2G).

Recently, a new definitive endoderm-specific desert lncRNA, *T-REX17* (Transcript Regulating Endoderm and activated by *SOX17*), has been identified during human endoderm differentiation. *T-REX17* is transcribed approximately 230 kb upstream of the *SOX17* locus and resides within the same topologically associating domain (TAD), a self-contained 3D chromatin region where DNA elements interact more frequently within the domain than with those outside. Surprisingly, CRISPR-dCas9-mediated silencing of *T-REX17* transcripts does not affect *SOX17* expression or chromatin occupancy. Instead, *T-REX17* expression is *SOX17*-dependent. During endoderm differentiation, *T-REX17* deletion leads to the upregulation of pluripotency-associated genes and the downregulation of endoderm and WNT pathway genes. Consequently, *T-REX17*-depleted cells lose their capacity to differentiate into endoderm and the derived pancreatic lineages. RNA-pulldown, followed by mass spectrometry analysis, reveals that *T-REX17* interacts with the ribonucleoprotein *HNRNPU*, though its detailed functional mechanism in endoderm differentiation remains unclear (Landshammer et al. 2023) (Fig. 2H).

In addition, our group recently reported another desert lncRNA, *HIDEN* (human IMP1-associated "desert" definitive endoderm lncRNA), which is crucial for endoderm differentiation. While *HIDEN* is not required to maintain pluripotency, its absence blocks definitive endoderm differentiation from human PSCs by modulating the WNT signaling pathway. Further investigation showed that *HIDEN* transcripts are primarily enriched in the cytoplasm of endoderm cells and interact with the RNA-binding protein IGF2BP1. Using transcriptomic analysis and RNA immunoprecipitation sequencing of IGF2BP1, the WNT receptor *FZD5* is identified as a target of the *HIDEN*/IGF2BP1 complex. *HIDEN* appears to regulate human endoderm differentiation by interacting with IGF2BP1 and stabilizing *FZD5* mRNA (Lu et al. 2023) (Fig. 2I).

Although desert lncRNAs exhibit higher expression levels and greater tissue/cell-specific expression compared to other types of lncRNAs (Fig. 1D, E), their functional and mechanistic studies remain limited. Furthermore, the fact that desert lncRNAs are located far from PCGs may help address the question of whether it is the lncRNA locus or its transcripts that perform the function. Additionally, the regulatory mechanisms of desert lncRNAs could provide new insights into evaluating the role of lncRNAs in biological processes.

## Conclusions and perspectives

Recent studies have revealed that lncRNAs play a crucial role in regulating proper endoderm differentiation by modulating the expression of endoderm-specific genes and critical signaling pathways (Fig. 2). Here we categorize lncRNAs into three distinct groups and summarize current research on their roles during endoderm differentiation. By categorizing lncRNAs into overlapped, proximal, and desert subgroups, this approach facilitates a more convenient investigation of their regulatory mechanisms. Overlapped and proximal lncRNAs exhibit strong potential for cis-regulatory effects on adjacent PCGs, whereas desert lncRNAs may primarily function *in trans* by interacting with RNA-binding proteins (RBPs), engaging in other long-range regulatory mechanisms. Given these distinct modes of action, experimental approaches such as RNA pulldown followed by mass spectrometry and chromatin isolation by RNA purification (ChIRP) are particularly suitable for studying desert lncRNAs. Unlike traditional classifications based on function, length, biogenesis pathways, or subcellular localization—which add complexity and may be challenging for researchers new to the field, this simplified classification offers a clearer, more accessible framework for understanding lncRNA regulatory mechanisms within a genomic context. Additionally, it enables systematic comparisons of their protein-coding potential, expression levels, subcellular localization, and regulatory roles across various biological processes. By applying this framework, we can gain deeper insights into the distinct functional properties of lncRNAs and their contributions to gene regulation. Furthermore, this classification lays the groundwork for future research, helping bridge gaps in our understanding of lncRNA-mediated regulatory networks.

LncRNAs typically exhibit low expression levels, a tendency for nuclear localization, and cell-type-specific expression compared to PCGs. Among these, overlapped lncRNAs are the most abundant and have been extensively studied due to their close association with PCGs across various biological processes. Most of the overlapped lncRNAs function by regulating their neighboring genes *in cis* during endoderm differentiation, such as *GATA6-AS1* and *Evx1as*. However, it is not always the case. In contrast, the overlapped lncRNA *FOXD3-AS1* does not appear to interact with the neighboring gene *FOXD3* in the context of definitive endoderm differentiation. The proximal lncRNAs, such as *DEANR1*, *DIGIT*, and *Halr1*, exhibit more complex regulatory mechanisms during differentiation. While these lncRNAs are located near PCGs, they can also be distantly positioned within the genome and influence endoderm differentiation by looping chromatin to promote interactions between regulatory elements and the promoters of target genes. The roles and mechanisms of desert lncRNAs in endoderm differentiation remain less understood due to their diverse modes of action and unpredictable targets. For instance, *LINC00458* and *T-REX17* interact with SMAD2/3 and HNRNPU, respectively, to promote endoderm differentiation, yet their precise mechanisms remain unclear. Further investigation requires integrating multi-omics approaches, such as RNA immunoprecipitation sequencing (RIP-seq) and chromatin isolation by RNA purification sequencing (ChIRP-seq), to map their direct targets and interaction networks. Notably, *T-REX17* deletion leads to the upregulation of JUN pathway genes, and inhibition of JNK hyperactivity with the inhibitor XVI partially rescues the differentiation defect in *T-REX17*-depleted cells, suggesting that the JUN pathway is a potential target of the *T-REX17*-HNRNPU axis in endoderm differentiation. Previous studies indicate that HNRNPU plays key roles in nuclear matrix organization and splicing through interactions with lncRNAs (Hacisuleyman et al. 2014; Zhang et al. 2024), providing possible mechanistic insights into *T-REX17* function. Additionally, the cytoplasmic desert lncRNA *HIDEN* functions by interacting with IGF2BP1 to stabilize the mRNA of the WNT signaling receptor *FZD5*, thereby regulating endoderm differentiation. These evidences are consistent with the fact that lncRNAs play crucial roles in epigenetic and transcriptional regulation (e.g., *EVX1as*, *GATA6-AS1*, *DEANR1*, and *Halr1*), scaffolding biomolecular condensates (e.g., *DIGIT*), and post-transcriptional regulation (e.g., *HIDEN*) (Chen and Kim 2024) (Table 1).

**Table 1:** The summary of lncRNAs in endoderm differentiation

Classification	Name	LncRNA function	References
**Overlapped lncRNAs**	*Evx1as*	*Evx1as* RNA binds chromatin and interacts with transcription coactivators to enhance *EVX1* expression and regulate endoderm differentiation	Luo et al. 2016
	*GATA6-AS1*	*GATA6-AS1* interacts with SMAD2/3 to promote *GATA6* expression and facilitate mesendoderm differentiation	Jha et al. 2020; Yang et al. 2020
	*FOXD3-AS1*	Cytoplasmic *FOXD3-AS1* interacts with E3 ubiquitin ligase to prevent phosphorylated β-catenin degradation, modulating pluripotency and differentiation	Guo et al. 2020; Haswell et al. 2021
**Proximal lncRNAs**	*DEANR1*	*DEANR1* interacts with SMAD2/3 and facilitates SMAD2/3 recruitment to endodermal gene *FOXA2* promoter via chromatin looping	Jiang et al. 2015
	*DIGIT*	*DIGIT*, regulated by a SMAD2/3-occupied enhancer, interacts with BRD3 to bind H3K18ac and form phase-separated condensates, regulating endodermal gene expression	Daneshvar et al. 2020, 2016
	*Halr1 (linc1547,* *linc-Hoxa1,* *Haunt)*	*Halr1* is induced by RA signaling, with *Hoxa1* binding to enhancer regions to activate its expression. In turn, *Halr1* acts as a scaffold to repress *Hoxa1* by orchestrating interactions between multiple enhancers and *Hoxa1* chromatin. In the other hand, *Halr1* can also recruit PURB to repress *Hoxa1* expression *in cis*	Guttman et al. 2011; Maamar et al. 2013; Su et al. 2021; Yin et al. 2015
**Desert lncRNAs**	*LINC00458*	Soft substrate-induced *LINC00458* interacts with SMAD2/3, promoting endodermal lineage specification with unclear mechanism	Chen et al. 2020
	*T-REX17*	*T-REX17* resides within the same TAD as *SOX17* but functions independently. It is crucial for DE differentiation, although its mechanism remains unresolved	Landshammer et al. 2023
	*HIDEN*	*HIDEN* interacts with IGF2BP1 to stabilize *FZD5* mRNA, thereby regulating DE differentiation	Lu et al. 2023

LncRNA function is underestimated due to limited research approaches. Early studies mainly focus on individual lncRNA or functional screening of lncRNAs in small-scale. CRISPR-based library screening provides genome-wide intervention of lncRNAs in various cells. Recently, transcriptome-scale RNA-targeting CRISPR screens reveal 46 universally essential and 778 context-specific essential lncRNAs in human cells (Liang et al. 2024). In addition, genome-wide CRISPR screening and RNA-seq also have identified numerous lncRNAs that are differentially expressed between ESCs and definitive endoderm cells, but with their roles still largely unknown (Haswell et al. 2021; Lu et al. 2023). In vitro studies have demonstrated the critical roles of lncRNAs in regulating pluripotent state transitions and lineage differentiation (Lu et al. 2021). However, due to restricted access to human embryonic materials and ethical concerns, the precise functions of lncRNAs in early human embryos remain largely unexplored, presenting significant challenges for developmental research. Recent advancements in human blastoids may offer a promising platform for studying lncRNA functions in a model that closely mimics in vivo conditions (Liu et al. 2021; Yu et al. 2023; Yu et al. 2021).

Another major challenge in investigating lncRNA function lies in their multifaceted, complex, and often elusive roles in biological processes. LncRNAs can regulate epigenetic, transcriptional, and post-transcriptional processes or act as scaffolds in different contexts (Chen and Kim 2024). A single lncRNA may function through its RNA transcript, genomic locus, or even by encoding a small peptide, and in some cases, multiple regulatory mechanisms coexist. For instance, lncRNA *Halr1* regulates precise Hoxa1 expression by orchestrating interactions between the *Hoxa1* promoter and enhancers within its locus (Su et al. 2021; Yin et al. 2015). These complexities necessitate the use of diverse methods to study lncRNA function and mechanisms. Current approaches to lncRNA loss-of-function studies include: (1) RNA-induced silence complex (RISC) mediated lncRNA cleavage, such as small interfering RNA and shRNA, are preferred for suppressing cytoplasmic lncRNAs; (2) antisense oligonucleotide (ASO) mediated knockdown, such as LNA gapmers, forms DNA-RNA duplexes and induces RNaseH-mediated cleavage, effectively targeting nuclear lncRNAs; (3) poly(A) and CRISPRi mediated lncRNA transcription inhibition, are used to determine whether transcription initiation, rather than RNA transcripts, drives lncRNA function; (4) CRISPR-Cas9 mediated genomic deletion of lncRNA gene body or promoter, is used to inhibit lncRNA expression thoroughly but may destroy the regulatory elements within lncRNA locus; (5) CRISPR-Cas13 mediated lncRNA knockdown, making it suitable for specially studying transcript-dependent functions (Lennox and Behlke 2016; Liu and Lim 2018; Yip et al. 2021). For lncRNA *Halr1*, Maamar and colleagues observed that ASO-mediated knockdown, but not siRNA, increased Hoxa1 expression, despite achieving similar overall *Halr1* knockdown efficiency. This difference is likely due to the limited effectiveness of siRNA in targeting nuclear lncRNAs (Maamar et al. 2013). Yin and colleagues further discovered that large-fragment deletion of the *Halr1* locus caused effects on *Hoxa1* expression opposite to those observed with *Halr1* transcript depletion. This difference was attributed to the presence of enhancer elements within the *Halr1* locus (Yin et al. 2015). Additionally, lncRNA overexpression or rescue differs from that of protein-coding genes. For lncRNAs functioning *in cis*, in situ overexpression is essential, requiring tools like CRISPR knock-in or CRISPR-activation systems. This underscores the importance of selecting appropriate methodologies tailored to lncRNA localization and function.

Although lncRNAs are defined by their low protein-coding potential, accumulating evidence has revealed functional peptides encoded by lncRNAs, particularly in cancer cells (Choi et al. 2019; Xing et al. 2020), adding complexity to their functions and regulatory mechanisms. LncRNAs can produce small peptides from small or short open reading frames (sORFs). Based on this characteristic, researchers have developed algorithms to predict sORFs in lncRNA transcripts, along with user-friendly prediction tools (Xing et al. 2020). Combining ribosome profiling with mass spectrometry has enabled global screening of lncRNA-encoded peptides (Plaza et al. 2017). Recently, Zheng and colleagues used ribosome profiling coupled with CRISPR/Cas9 screening to identify lncRNA-encoded ORFs in human cancer (Zheng et al., 2023). In endoderm and its derived pancreatic progenitor cells, Gaertner and colleagues identified 625 novel sORFs within lncRNAs, with a median peptide length of 47 amino acids (Gaertner et al. 2020). Among these, the knockdown of *DEANR1* impaired endocrine cell differentiation. However, the individual disruption of *DEANR1*’s sORFs did not affect this process, suggesting potential redundancy in the functions of *DEANR1*’s microproteins and sORFs (Gaertner et al., 2020). Furthermore, lncRNA-encoded peptides are also functional in the muscle system. Myoregulin (MLN), a putative lncRNA-encoded micropeptide, regulates muscle performance (Anderson et al. 2015), while the polypeptide SPAR, encoded by *LINC00961*, is crucial for regulating mTORC1 and muscle regeneration (Matsumoto et al. 2017). Additionally, another class of lncRNAs, circular RNAs (circRNAs), has been shown to undergo pervasive translation driven by short internal ribosome entry site (IRES)-like elements (Fan et al. 2022; Yi et al. 2024). Numerous circRNAs are expressed and play critical roles in regulating stem cell differentiation (Lin et al. 2021). For instance, Yu and colleagues demonstrated that *circBIRC6* and *circCORO1C* are functionally linked to the pluripotency maintenance of human ESC and reprogramming. Mechanistically, *circBIRC6* is enriched in the RISC complex, acting as a microRNA sponge to promote the pluripotent state (Yu et al. 2017). However, the functional roles of peptides translated from circRNAs during early embryonic development remain largely unexplored.

## Data Availability

Not applicable.

## Abbreviations

ASO: Antisense oligonucleotide
ChIRP: Chromatin isolation by RNA purification
circRNAs: Circular RNAs
CRISPRi: CRISPR interference
ESCs: Embryonic stem cells
FISH: Fluorescent in situ hybridization
ICM: Inner cell mass
iPSCs: Induced pluripotent stem cells
IRES: Internal ribosome entry site
LNAs: Locked nucleic acids
lncRNAs: Long noncoding RNAs
LSEC: Liver sinusoidal endothelial cell
PCGs: Protein-coding genes
PSCs: Pluripotent stem cells
RA: Retinoic acid
RBP: RNA-binding protein
RIP: RNA immunoprecipitation
RISC: RNA-induced silence complex
shRNAs: Short hairpin RNAs
sORFs: Small or short open reading frames
TAD: Topologically associating domain
